# Fast track sacrospinous ligament fixation: subjective and objective outcomes at 6 months

**DOI:** 10.1186/s12905-021-01309-1

**Published:** 2021-04-16

**Authors:** Susanne Greisen, Susanne Maigaard Axelsen, Karl Møller Bek, Rikke Guldberg, Marianne Glavind-Kristensen

**Affiliations:** 1 Department of Obstetrics and Gynecology, Aarhus University Hospital, Aarhus, Denmark; 2 Regionshospitalet Horsens, Sundvej 30, 8700 Horsens, Denmark

**Keywords:** Apical prolapse, Fast-track surgery, Local anesthesia, Sacrospinous ligament fixation (SSLF)

## Abstract

**Background:**

Sacrospinous ligament fixation (SSLF) is a widely used vaginal procedure for correction of apical vaginal prolapse. The objective of this study was to evaluate subjective and objective outcomes of SSLF performed in a fast-track setting.

**Methods:**

This was a prospective cohort study of sacrospinous ligament fixation performed using local anesthesia and light sedation in a fast-track setting at Aarhus University Hospital between April 2016 and December 2017. Objective signs of prolapse were assessed by gynecological examination preoperatively and at 6 months after the operation. Subjective symptoms were evaluated by questionnaires (the Pelvic Floor Distress Inventory (PFDI 20), and the Patient Global Impression of Improvement (PGI-I) supplemented with individual questions from the ICIQ-vaginal Symptoms (ICIQ-VS) and Sexual Questionnaire-IR (PISQ-12) questionnaires).

**Results:**

One hundred and three women with a median age of 65 (36–84) years were included. Previous hysterectomy had been performed in 40% of the women, and 43% had a history of previous prolapse operations. At follow-up, 75% of the women had apical descent less than stage 2. However, 18% had anterior vaginal wall prolapse beyond the hymen, and 25% had recurrence of the apical prolapse stage 2 or more and were offered reoperation. Bladder and anal symptoms improved in most women after the operation, and the number of women reporting dyspareunia was halved. In the overall assessment by Patient Global Impression of Improvement (PGI-I) questionnaire, 76% reported improvement. No serious operative complications were reported, and 81% of the patients were discharged on the day of the surgery.

**Conclusion:**

In this cohort with a high rate of previous prolapse surgery, sacrospinous ligament fixation performed in a fast-track setting showed subjective and objective results comparable to the results of apical native tissue repair reported in the literature. Furthermore, the complication rate was low.

*Trial registration *This study was notified to The Central Denmark Region Committees on Health Research Ethics on July 7, 2015, and was approved by The Danish Data Protection Agency (1-16-02-442-15). All methods were performed in accordance with the relevant guidelines and regulations. An informed consent for participation in the study and acceptance of using data for scientific purposes and publication was signed by all patients.

## Background

Surgical correction of symptomatic vaginal prolapse is performed in 12% of all women by the age of 80 [1]. The sacrospinous ligament fixation (SSLF), developed by Richter in 1958 [2], is one of the most widely used vaginal procedures for correction of apical prolapse. The procedure can be used both for vaginal vault fixation after hysterectomy and for hysteropexy with preservation of the uterus. While anterior vaginal wall prolapse is the most common site of prolapse, loss of apical support is usually present in women with prolapse that extends beyond the hymen [3]. There is growing recognition that adequate support for the vaginal apex is also an essential component of durable surgical repair for advanced prolapse in the anterior and posterior compartment [3].

The use of less invasive surgical techniques, suture delivery devices and local anesthesia with sedation, has made it possible to perform SSLF as a fast-track procedure, with the patient being discharged on the day of the operation [4].

The objective of this study was to evaluate both subjective and objective outcomes of fast-track SSLF in a population with a high incidence of previous prolapse operations at 6-month follow-up.

## Methods

This was a prospective cohort study of 103 women who underwent SSLF at Aarhus University Hospital, Aarhus, Denmark, between April 2016 and December 2017. All women offered an SSLF for either apical prolapse or for anterior or posterior prolapse with a pronounced apical component were included in the study if they could read Danish and accepted participation. Previous prolapse operations were not an exclusion criterion nor were other medical conditions.

Preoperatively, all women had bulge and/or dragging symptoms once a week or more. All women had objective findings of either predominantly vaginal vault/uterine prolapse or predominantly anterior or posterior vaginal wall prolapse with a pronounced apical component, defined and staged according to the Pelvic Organ Prolapse Quantification (POP-Q) System as stage 2 or more in at least one compartment [5].

In addition to apical repair concomitant anterior repair, posterior repair or both were performed when needed. Concomitant incontinence surgery was not performed.

### Operation technique

The operation was performed in a day surgery unit. Standardized requirements for antiseptic procedures were respected. The skin of the vulva was disinfected with 0.5% chlorhexidine in 85% ethanol. Right-side SSLF of the vaginal apex was performed in all the women. They had an indwelling catheter in the bladder during the operation. Antibiotics were not routinely used. All women had a bilateral pudendal block, infiltration anesthesia and light sedation as described in a previous publication [4]. The vaginal mucosa was opened in the midline of either the anterior or the posterior vaginal wall from the vault/cervix to 2 cm from the external urethral meatus or in the posterior vaginal wall to the hymen. Blunt dissection was performed to access the paravaginal space. This dissection was narrow, allowing only two fingers to reach the ligament to minimize the surgical trauma. Digitally, the sacrospinous ligament was identified and two polydioxanone 0–0 sutures were placed approximately 1.0 and 2.0 cm medial to the ischial spine with the Capio™ Suture Capturing Device. Defects in or attenuation of the pubocervical or rectovaginal fascia were repaired by duplication of the fascia with a continuous polydioxanone 3–0 suture. Sacrospinous ligament fixation sutures were attached to the apical part of either the anterior or posterior vaginal wall fascia and to the Cardinal ligaments if the uterus was preserved. The vaginal wall mucosa was trimmed and closed with a running polyglactin 3–0 suture. After the operation, a cystoscopy was done to visualize free passage of urine from the ureters. Afterwards, the vagina was packed. The catheter and the vaginal pack were removed after 3 h. Operating time, intraoperative complications, postoperative complications, and hospital stay were recorded for all women.

If the woman had an acceptable pain score and was able to empty her bladder, she was discharged on the day of the operation. If she had bladder emptying problems, she was admitted to the patient hotel where a nurse would perform intermittent catherization. If the woman had problems such as pain, nausea or vomiting she was admitted to the stationary ward until the problems subsided.

Detailed data on hospital stay and patient satisfaction with the anesthesia during the operation have been reported previously [4].

### Questionnaires

The Pelvic Floor Distress Inventory (PFDI-20) (Validated Danish version), and individual questions from the ICIQ-Vaginal Symptoms (ICIQ-VS) and Pelvic Organ Prolapse/urinary Incontinence Sexual Questionnaire, IUGA-Revised (PISQ-12) questionnaires were answered by all women before surgery and at 6-month follow-up. Patient Global Impression of Improvement (PGI-I) symptom questionnaire was answered at follow-up.

### Statistical analysis

Patient characteristics were reported as medians (range) or frequency (percent) as appropriate. To account for missing data, subjective data are presented as the fraction of the total number answering the specific question. Pre and postoperative data were compared using the Chi-square test. A *p* value less than 0.05 was considered statistically significant. Analyses of secondary outcomes were considered exploratory and *p* values provided for descriptive purposes only.

## Results

One hundred and three women were included in the study. Baseline characteristics are shown in Table 1. Forty-one (40%) women had previous hysterectomy and 44 women (43%) had undergone surgery for prolapse without hysterectomy. The majority of these previous operations were in the anterior compartment. One woman did not attend the clinical examination at 6-month follow-up and one woman did not answer the questionnaires. Thus, data from 102 women were available for analysis of the subjective and objective results.

**Table 1:** Baseline characteristics of 103 women recruited for the prospective follow-up study of sacrospinous ligament fixation in a fast-track setting

Baseline characteristics	
Age (years)	65 (36–84)
BMI (kg/m^2^)	26 (18–38)
Menopausal status (age > 52 years)	87 (85%)
No previous hysterectomy or POP operation	33 (32%)
Previous hysterectomy	41 (40%)
POP operations excluding hysterectomy	45 (43%)
1 previous POP operation	24 (23%)
2 previous POP operations	18 (17%)
3 previous POP operations	2 (2%)

*BMI* body mass index, *POP* pelvic organ prolapse

Data are presented as median (range) or number (percent)

Preoperatively, 18 women had stage 1, 68 had stage 2, 15 had stage 3, and 2 women had stage 4 apical prolapse. Preoperative POP-Q scores are shown in Table 2 whereas subjective data are listed in Table 3.

**Table 2:** Anatomical findings regarding degree of prolapse (POP-Q score) in women who had undergone sacrospinous ligament fixation. Preoperative findings and results 6 months after operation

	Preoperatively	Postoperatively
Aa	0 (− 3 to + 6)	− 1 (− 3 to + 3)
Ba	+ 1 (− 3 to + 6)	− 1 (− 5 to + 3)
C	0 (− 3 to + 7)	− 4.5 (− 10 to + 3)
Ap	− 1.5 (− 3 to + 3)	− 2 (− 3 to + 3)
Bp	− 2 (− 5 to + 4)	− 2 (− 6 to + 3)
TVL	7 (3 to 10)	8 (5 to 10)

Data are presented as median (range)

**Table 3:** Subjective findings before and 6 months after the sacrospinous ligament fixation

	Preoperative	Postoperative	P-value
Vaginal bulge	94% (94/100)	47% (47/100)	< 0.001
Urinary frequency	58% (58/100)	40% (39/96)	0.015
Urge incontinence	54% (53/99)	45% (46/103)	0.205
Stress incontinence	44% (44/99)	33% (32/97)	0.100
Anal straining	37% (36/97)	25% (24/96)	0.071
Insufficient Anal emptying	48% (48/98)	39% (37/95)	0.163
Anal incontinence	23% (23/98)	15% (14/96)	0.116
Sexually active	40% (40/100)	45% (41/91)	0.482
Sexually inactive due to vaginal bother	31% (19/60)	10% (5/49)	0.007
Pain at intercourse (sometimes or often)	34% (18/53)	17% (8/48)	0.045
Reduced sensitivity in the vagina	18% (18/98)	7% (6/90)	0.010
Vaginal soreness	27% (27/99)	16% (16/97)	0.067

Data are presented as percent (number of women with the symptom/number of women who answered the specific question). Pre and postoperative data were compared using the Chi-square test

In addition to apical repair, concomitant anterior repair (n = 69), posterior repair (n = 24) or both (n = 5) was performed when needed. The median operating time was 40 (range 20–90) minutes.

The majority of the 102 women (81%) were discharged on the day of the operation.

### Complications

There were no serious operative complications, no visceral injury, and no peri-operative blood transfusion were given. Postoperatively, 11 women were examined in the outpatient clinic due to minor complications. Four women were readmitted. One due to a more serious complication. Postoperative complications are described in Table 4.

**Table 4:** Complications after sacrospinous ligament fixation in local anesthesia with light sedation in a fast-track setting

Admitted to the ward on the day of the operation	Number of women
Complications to the pudendal block	
Short term sensory disturbance in the right leg	5
Short term urinary retention	3
Bleeding (no intervention)	1
Nervousness	1
Re-admitted after discharge	
Wound infection	3
Sepsis	1
Urinary retention	1
Seen in the outpatient clinic after discharge	
Wound infection	4
Disturbed vaginal sensation	1
Persisting buttock pain (< 6 month)	1
Persisting urinary retention (< 3 weeks)	2

Data are presented as number of women

### Anatomical results

At 6-months follow-up, 75% (77/102) had apical descent less than stage 2 (48/102 grade 0, 29/102 grade 1). The median change in POP-Q score for the point which represents the cervix/vault changed from 0 to − 4.5 at 6-month follow-up and the total vaginal length was preserved (Table 2). At the time of follow-up, 18% (18/102) had anterior vaginal wall prolapse beyond the hymen 87% of these were recurrent anterior prolapse. Twenty-five women had recurrent apical prolapse stage 2 or more. Twenty-four women were offered an operation for recurrence of apical prolapse. One woman preferred treatment with a vaginal pessary. There was no significant difference in recurrence rate between women with or without a uterus (*p* = 0.064).

### Subjective results

The sensation of vaginal bulge was significantly reduced after the operation (*p* < 0.001). Prolapse symptoms were reported in four categories: no bother (n = 58), a little bother (n = 17), some bother (n = 7), and a great deal of bother (n = 20). Thus, at follow-up 26% (27/102) of the women still felt some or a great deal of bother from a vaginal bulge.

Bladder emptying, urinary frequency, urgency and stress incontinence all improved slightly. However, only improvement of urinary frequency was statistically significant (*p* = 0.015) (Table 3). Anal symptoms decreased after the operation. Thus, less women experienced straining, difficult bowel emptying or anal incontinence after the operation (Table 3).

The percentage of women who reported being sexually active before the operation was 40% (40/100). After the operation this number was 45% (41/91). Moreover, among the women reporting being sexually inactive prior to the operation, 31% (19/60) declared that this was due to vaginal bother. After the operation, only 10% (5/49) reported vaginal bother as the cause of sexually inactivity. After SSLF, the number of women reporting dyspareunia was halved (Table 3).

In the overall quality of life assessment (PGI-I), 76% (64/84) reported improvement (little, much or very much better) 6 months after SSLF. No change was reported by 17%, and 7% felt worse than before the operation. Patient reported goals with the operation were achieved by 60% (50/83) (completely or a great deal).

## Discussion

This prospective study of SSLF in a population with a high incidence of previous operations for prolapse showed that 75% of the women had a good objective result, with apical descent less than grade 2 six months postoperatively. Moreover, 74% of the women felt little or no bother because of vaginal bulging. Urinary, anal and sexual symptoms improved slightly in most women. However, 24 women were offered re-operation due to recurrence of the apical prolapse.

Most studies on operations for pelvic organ prolapse (POP) include only women with no history of prolapse surgery. Prior POP operations are known to decrease success rates after subsequent prolapse operations substantially [6–8]. This makes the population in the present study a “high risk” population, since 42% had previous POP surgery without hysterectomy and 40% had previous hysterectomy.

In this study, the women underwent surgery in a fast-track setting that was made possible by using a suturing device to minimize the surgical trauma by avoiding wide dissection of the paravaginal space to attend the sacrospinous ligament. Furthermore, general anesthesia was replaced by a combination of pudendal block, infiltration anesthesia and light sedation, ensuring fast recovery of the woman after surgery. Due to these changes, 81% of the women could return to their home on the day of surgery. In a previous study, we reported a very high patient satisfaction with the anesthesia procedure [4].

A Swedish study, comparing SSLF performed with a suturing device to traditional SSLF showed that recurrence of symptoms at 1-year follow up was slightly more common with the minimally invasive technique. Thus, 28% had prolapse symptoms in the suturing device group compared to 21% in the traditional group. However, patient satisfaction was similar in the two groups [9]. Other studies report objective success rates between 67 and 95% when suturing devices are used for SSLF, making the results of these techniques comparable to traditional techniques [10, 11].

In the present study, 25% of the women had apical prolapse stage 2 or more at 6-month follow-up. Using the criterion of an objective prolapse stage 2 or more, Morgan et al. in a systematic review of SSLF found that failure rates were 21.3% in the anterior compartment, 7.2% in the apical compartment, and 6.3% in the posterior compartment [12]. The pooled failure rate regarding symptoms of prolapse was 10% (95% confidence interval 4–16%) [12]. A RCT comparing sacrospinous hysteropexy and vaginal hysterectomy showed that after sacrospinous hysteropexy, 100% had prolapse less than stage 2 and no prolapse symptoms at 12-months follow-up. However, when looking solely at anatomic outcomes in any compartment, 50% had stage 2 prolapse or greater, mostly due to anterior vaginal wall recurrence [13]. Another RCT comparing vaginal hysterectomy to sacrospinous hysteropexy (SSHP) found that 79% of the women in the SSHP group had anatomic findings of prolapse less than stage 2 at 12-months follow-up [14]. The rather high recurrence rate in our data compared to the RCTs might be due to the different populations, since both RCTs included only patients undergoing primary surgery. Moreover, in both RCTs non-absorbable sutures were used for the sacrospinous suspension whereas we used absorbable polydioxanone sutures. We chose to use absorbable sutures to minimize the risk of persisting buttock pain which is a known complication to SSLF. We found no patients with buttock pain after 6 months, but use of absorbable sutures might be part of the explanation for the high recurrence rate.

Uterosacral ligament suspension (ULS) is another native tissue repair technique for suspension of the vaginal vault after hysterectomy. A RCT study (OPTIMAL) compared ULS with SSLF and reported that an objective success rate of 60% was achieved with both techniques at 2-year follow-up. The study had very strict anatomical criteria for success, namely no apical descent greater than one-third into the vagina or anterior or posterior vaginal wall prolapse beyond the hymen. The study demonstrated that in the SSLF group, 13% of the women had anterior vaginal wall prolapse beyond the hymen after 2 years [15]. In the present study, 18% had anterior vaginal wall prolapse at 6-month follow-up. We previously published a retrospective study evaluating results of ULS that included women with a high rate of previous prolapse operations similar to that in the present cohort. Seven years after ULS, 35% were re-operated due to recurrent prolapse. Only 50% of the patients had recurrent apical prolapse but they did have recurrent prolapse in other compartments [16].

Only a few studies reporting on SSLF focus on subjective symptoms and functional results. In a register-based study from Sweden including 353 women undergoing SSLF, 18% still had bulge symptoms 1 year after surgery [9]. The OPTIMAL trial found that 18% had bothersome bulge symptoms 2 years after surgery [15]. In the present study, we found that 26% of the women still felt some or a great deal of bother caused by a vaginal bulge 6 months after surgery. In contrast to our study, less than 10% of included women in the OPTIMAL study had prior prolapse surgery. Moreover, permanent sutures were used for the suspension.

In the overall quality of life assessment PGI-I, 76% were improved 6 months after the surgery. Quality of life reflects the result of the surgery. Even though the operation might be an objective success, it may be followed by new symptoms affecting quality of life. Thus, a prospective study with 79 women undergoing prolapse surgery demonstrated that 72% found their situation improved after prolapse surgery while 7% felt worse at 3-month follow-up [17]. These findings are in accordance with our findings; however, in our study the lack of satisfaction might be due to failure of the repair.

The data on sexual behavior and symptoms showed no change in the proportion of women being sexually active before and after the operation. However, the sexually active women reported significantly better vaginal sensitivity, less soreness and less pain during intercourse compared to before the operation. These results are in accordance with previous findings, most patients reporting sexual function as improved or unchanged [18, 19].

Data on urinary symptoms after POP surgery are conflicting. A study investigating new pelvic symptoms after POP surgery showed that 25% have de-novo urgency and 23% have de-novo increased frequency [17]. However, other data demonstrate resolution of urgency in 62% of the women after POP surgery [20]. In our study, urinary frequency was significantly improved, whereas urinary incontinence symptoms were slightly improved after the operation.

Serious complications associated with sacrospinous fixation are infrequent but include neurovascular injuries. A review including 1229 patients found life-threatening hemorrhage in 0.2% of patients. Transfusion rate was 2% [21]. In the present study, no blood was given during the surgery and there was no visceral injury. The most common perioperative complications were short term urinary retention (5%). Only 2% were in the need of intermittent catherization (maximum 3 weeks). The short-term urinary retention might be ascribed to the pudendal block or the fact that the catheter was removed already three hours after the operation. The long-term retention in some women may have been due to the concomitant anterior colporrhaphy. Urinary retention after prolapse surgery is reported to range from 29 to 32% in the literature [12, 22]. Eight women in our study had postoperative wound infection. Only one woman had a serious infection. Others have reported infectious morbidity due to fever or abscess in 4% of patients after SSLF [12]. Antibiotics were not used routinely during the operation in our study. Despite this, the infection rate was low and acceptable.

This study has several limitations. It was an observational study with limited size and with a relatively short follow-up period. The study included both women with a primary prolapse operation and women with one or more previous prolapse operations. Moreover, both sacrospinous suspension of the vaginal vault and sacrospinous hysteropexy were included. The PISQ-12 questionnaire that we used was translated into Danish by the Danish Urogynecological Society, but the Danish version has not been validated. However, the study involved a systematic follow-up with both objective and subjective assessment and it reflected routine treatment in a standard care setting.

## Conclusion

In this cohort with a high rate of previous prolapse repair, SSLF performed in a fast-track setting showed subjective and objective results comparable to results of apical native tissue repairs reported in the literature. The complication rate was low.

## Data Availability

All data generated during this study are included in this published article and in a previous published study from our team [4]. The dataset used during the current study are available from the corresponding author on reasonable request.

## Abbreviations

ICIQ-VS: ICIQ-vaginal Symptoms
PFDI 20: The Pelvic Floor Distress Inventory
PGI-I: Patient Global Impression of Improvement
PISQ-IR: Sexual Questionnaire-IR
SLFF: Sacrospinous ligament fixation
ULS: Uterosacral ligament suspension
